# Transscrotal transverse incision for the treatment of middle and low cryptorchidism in children: experience from 796 cases

**DOI:** 10.1186/s12893-020-00710-1

**Published:** 2020-03-17

**Authors:** Yun-jin Wang, Liu Chen, Qi-liang Zhang, Yu Lin, Xu Cui, Jian-cai Chen, Chao-ming Zhou

**Affiliations:** Department of Pediatric Surgery, Fujian Maternity and Child Health Hospital, Fuzhou, 350001 People’s Republic of China

**Keywords:** Children, Low and middle cryptorchidism, Transscrotal incision, Testicular descent fixation

## Abstract

**Background:**

The purpose of this study was to summarize our clinical experience with transscrotal transverse incision in the treatment of low and middle cryptorchidism in children.

**Methods:**

A total of 796 children with low or middle cryptorchidism participated in this study from March 2012 to May 2018. Transscrotal transverse incision was used to treat low and middle cryptorchidism. Symptoms and signs were followed up at 1 week, 1 month, 3months and every six to 12 months thereafter.

**Results:**

Testicular descent fixation through transverse scrotal incision was successfully performed in all 796 children. All patients were discharged 1–2 days after the operation. During hospitalization and follow-up, 35 patients had complications, including 7 cases of cryptorchidism recurrence, 5 cases of poor scrotal incision healing, and 23 cases of scrotal haematoma. There were no complications, such as bladder injury, testicular atrophy, inguinal hernia or hydrocele.

**Conclusion:**

Transscrotal transverse incision is a safe and feasible method for the treatment of middle and low cryptorchidism. It has the advantages of less trauma and an aesthetic scar after operation.

## Background

Cryptorchidism is the most common congenital malformation of the urinary and reproductive system in children. The incidence of cryptorchidism is 3–5% [1]. Complications such as torsion, injury, malignant change, decreased fertility and even infertility can occur in cryptorchidism [2–7]. Therefore, early active treatment is needed. Surgery is the main treatment for cryptorchidism. After Annandale successfully performed orchidopexy in 1879 [8], testicular descent fixation through an inguinal incision became a classic method for the treatment of cryptorchidism of the inguinal type and the supracostal type. However, there were obvious surgical scars in the inguinal area after the operation, which affected the patients’ appearance. With the continuous progression of people’s requirements for surgery, minimally invasive surgery has become more popular. Transverse scrotal incision for cryptorchidism not only is safe, reliable and effective but also has the advantages of short operation time, more aesthetic scar after operation,and minimal invasiveness in terms of maintaining the intact anatomical structure of the groin. In recent years, transverse scrotal incision has been widely and successfully used in the treatment of cryptorchidism in various nations. However, for this surgical technique the selection of the patient is very important and if the testicle is present in the inguinal canal should have good mobility and traction toward the external inguinal ring and scrotum. We reviewed the clinical data of 796 children with low and middle cryptorchidism and summarized our clinical experience of 6 years.

## Materials and methods

This study was approved by the ethics committee of our university and strictly adhered to the tenets of the Declaration of Helsinki. All patients’ guardians signed an informed consent form before the operation.

### Patients

The clinical data of 796 cases of cryptorchidism from March 2012 to May 2018 were retrospectively analysed, including preoperative, intraoperative and postoperative data and follow-up data. The cryptorchidism below the inner ring of inguinal canal is called the middle and low cryptorchidism, and the cryptorchidism below the outer ring of inguinal canal is called the low cryptorchidism [9].

The inclusion criteria were medium or low cryptorchidism. Patients were excluded from this study if they had other types of cryptorchidism, such as abdominal cryptorchidism; had complications from other diseases requiring simultaneous surgical treatment, such as hypospadias, inguinal hernia, and hydrocele; or refused to sign the consent form or adhere to the follow-up schedule.

According to the clinical manifestations, physical examination and ultrasonography, the diagnosis of cryptorchidism was clear in all patients, including 243 cases on the left, 463 cases on the right, and 90 cases on both sides. Their ages were 10 months to 5 years (Table 1). Ultrasonography not only can help further clarify the diagnosis and testicular location, but also provides the exact data of testicular size, which was useful for postoperative follow-up to evaluation of testicular development. Routine clinical examinations, including standard lead electrocardiogram, chest radiography and blood examination, were performed before the operation to eliminate operative contraindications.

**Table 1:** Clinical data of the patients in this study

Item	
Number of patients	796
Age, median (range)	2 years (10 month-7 years)
Weight,median (range)	14 (9.5–25)kg
No. transcrotalorchidopexies	
Left side	243 (30.5%)
Right side	463 (58.2%)
Bilatera	90 (11.3%)
Operation time, median (range)	
Unilateral	20 (13-28 min)
Bilateral	41 (20-49 min)
Time of hospital stay, median (range)	1 (1–2)days
Duration of follow-up, median (range)	3.8 years (10 month-6.5 years)
Incision length, median (range)	1.6 (1.0–2.1)cm

### Technology

After induction of anaesthesia, the patient was placed in the supine position with the waist slightly raised using a cushion. We routinely disinfected and draped the surgical area. A 2.0-cm stria incision was made in the middle part of the scrotum. The skin and subcutaneous tissue were cut layer by layer. We fully exposed the external ring using a small retractor pulled upward through the incision after bluntly separating the space between the external oblique and Scarpa’s fascia. The aponeurosis of the external abdominal oblique muscle was cut 0.5 to 1.0 cm through the external ring, and the middle and lower segments of the groin were thus fully exposed. After pushing the testicle to the area near the outer ring and clamping and cutting the processus vaginalis, the testicle was visible. The tissue between the spermatic cord and processus vaginalis was bluntly and sharply separated until the testicle could be descended to the bottom of the scrotum without tension. We transected the processus vaginalis in a high position and reconstructed the outer ring orifice by suturing the aponeurosis of the external abdominal oblique muscle to ensure the integrity of the inguinal canal. The testicle was sutured in an interrupted manner to the dartos fascia of the scrotum with no torsion. We then closed the scrotal incision using 5–0 absorbable interrupted sutures.

For the patients with bilateral cryptorchidism, the incisionof one side was slightly below or above the other side.

## Results

A total of 796 children with cryptorchidism (including 243 cases on the left side, 463 cases on the right side and 90 bilateral cases) successfully underwent testicular descent fixation through transverse scrotal incision. The median operative time of unilateral and bilateral cryptorchidism was 20 (13–28) minutes and 41 (20–49) minutes, respectively. All patients were discharged 1–2 days after the operation (Table 1). During hospitalization and follow-up, there were 35 patients with complications, including 7 cases of cryptorchidism recurrence, 5 cases of them were in the root of scrotum and 2 cases of them were near the outer ring of inguinal canal, which were cured after testicular descent fixation through inguinal incision, and no recurrence occurred; 5 cases of poor healing of scrotal incision, all healed after dressing change; 23 cases of scrotal haematoma, without special treatment, all subsided 1–2 weeks after operation. There were no complications, such as testicular atrophy, inguinal hernia or hydrocele. The follow-up period ranged from 10 months to 6.5 years, with a median follow-up time of 3.8 years. The follow-up intervals were 1 week, 1 month and 3 months after the operation, then every six to 12 months. The content of the follow-up included the symptoms and signs of the children, which were followed up via outpatient visits.

## Discussion

The incidence of congenital cryptorchidism is 1–9% in full-term male infants and 30% in premature infants. Half of the testes can drop spontaneously within 3 months after birth, and 1% remain unchanged around the age of 1 [10, 11]. The testes of cryptorchidism children may continue to descend after birth, but the chance of self-descent after 6 months is significantly reduced. Therefore, children require evaluation for the possibility of a medical intervention after 6 months [12–15]. Surgery is the most important treatment for cryptorchidism.

With the increasing demands of children and their families regarding aesthetics after surgery, paediatric surgeons need to consider that minimally invasive surgery has become a trend in surgical operation, as patients demand not only that the surgery achieve the desired clinical effect but also that it achieve an aesthetically pleasing and scarless result [16]. At present, laparoscopic surgery and traditional open surgery are the main surgical methods. According to the location of the testis and physical examination, ultrasound can detect the testis to operate on. In recent years, laparoscopic surgery and transscrotal incision surgery have been widely used to treat cryptorchidism, and satisfactory clinical results have been achieved [17–19]. Laparoscopic testicular descent fixation without inguinal incision and inguinal canal incision involves less trauma, less pain and a good cosmetic effect. It can also fully free spermatic cord vessels to reach the lower pole of the kidney to ensure testicular tension-free descent into the scrotum. However, laparoscopic surgery requires laparoscopic equipment, which is costly and brings with it potential complications such as intestinal injury, bladder injury and subcutaneous emphysema caused by CO2 pneumoperitoneum [20, 21]. Furthermore the indications for laparoscopy are different from those of transscrotal surgery which is applied in palpable testicles, and the laparoscopic surgery is mainly used for abdominal cryptorchidism.

Because the scrotal skin of children is lax, the range of movement is large, and the distance between the outer ring orifice and the upper scrotum is short. Moreover, the length of the sheath process in most children with cryptorchidism is shorter than that in normal children. Therefore, the external inguinal ring can be exposed through scrotal incision, the sheath process can be transected or ligated at a high level, and the extraspermatic fascia and intraspermatic fascia can be completely transected to ensure sufficient release of the spermatic cord. Then, the testis can be placed in the scrotum without tension. In 1989, Bianchi et al. [22] reported that single-scrotal-incision testicular descent fixation (Bianchi) had the advantage of minimal invasiveness in the treatment of low cryptorchidism with testes that could be touched before operation, so it was gradually applied in the clinic [23]. Single-incision testicular descent fixation via the scrotum has the following advantages: First, the single incision is located in the scrotal fold, and there is no scar after operation [24]. Second, the operation time is short, the hospitalization time is short, and the postoperative recovery is fast. Third, there is no need to destroy the inguinal structure as in traditional surgery. It leads to less trauma, less pain and less bleeding after operation.

The key to the operation of transscrotal testicular descent fixation is that the extraspermatic fascia and intraspermatic fascia should be completely transected to ensure sufficient release of the spermatic cord and tension-free insertion of the testis into the scrotum. For older children, because the inguinal canal is relatively long, if it is difficult to fully release the spermatic cord vessels and ligate the sheath process at high position, the outer ring orifice can be cut by 0.5–1.0 cm, which can meet the needs of loosening the spermatic cord and ligating the sheath process. In this study, the outer ring orifice was cut in 31 cases. However, we believe that due to the difference in surgeons’ experience, if it is difficult to release spermatic cord vessels during the operation, even if the outer ring orifice is cut by 0.5–1.0 cm, testicular tension cannot be guaranteed to decrease, in which case they should convert to traditional surgery of groin incision. Takahashi et al. [25] considered that the excessive gap between the sarcolemma and the extraspermatic fascia in the process of the free spermatic cord was also an important cause of testicular retraction after operation. After narrowing the gap between the sarcolemma and the extraspermatic fascia during operation, no testicular retraction occurred in any case after operation. Therefore, we suggest that the gap between the sarcolemma and the extraspermatic fascia should be narrowed as far as possible during the operation to prevent testicular retraction. After the operation, 7 cases of testicular retraction were followed up. Among them, four cases were caused by insufficient spermatic cord freedom and too little tension at the time of fixation. In two cases, the spermatic cord was short, and there was little tension when fixed. Finally, one patient with poor scrotal development could not completely accommodate the testes. The blood supply of the testis is mainly composed of the testicular artery, vas deferens artery, cremaster vessel, testicular ligament and testicular vascular bed. Testicular descent fixation with preservation of the testicular ligament is of positive significance [26]. During the operation, attention should be paid to protecting the vas deferens, vas deferens and spermatic cord vessels to ensure a full testicular blood supply. The testicular blood supply should not be destroyed to pursue the descending position of the testis because this would cause testicular atrophy after operation [27]. According to the above principles, testicular atrophy did not occur in the follow-up of this study. There were 23 cases of scrotal haematoma in this study. The reason may be that operations on the scrotum takes a long time, create traction, entail some damage to the scrotum, and lead to swelling of the scrotum after operation. For these reasons, the operation should be gentle, attention should be paid to haemostasis, and the scrotum should be slightly compressed after surgery, all of which will help to reduce swelling.

Because of the short inguinal canal in children, the external inguinal ring can be exposed during the operation to achieve high ligation of the sheath process. According to our experience with laparoscopic surgery in the treatment of cryptorchidism, for patients without inguinal hernia or hydrocele before operation, the unclosed sheath process can also not be ligated, and only a high transverse section can be performed. It should be emphasized that a high cross-section must be achieved. The high-level transection of the sheath process is similar to laparoscopic incision of the peritoneum at the orifice of the inner ring, which can make the sheath process close itself in the orifice of the inner ring and avoid hidden dangers [28]. All patients were operated on accordingly, and no cases of inguinal hernia or hydrocele occurred in the follow-up after the operation.

Although this retrospective study had a large sample size there are still several limitations. First, this was a retrospective study with a limited number of patients from a single centre,and more research from multiple centres is needed to assess the effectiveness and complications of this technique. Second, the median follow-up duration was relatively short, and a longer follow-up period is warranted.

## Conclusions

Transscrotal transverse incision is a safe and feasible method for the treatment of middle and low cryptorchidism. It has the advantages of less trauma and an aesthetic incision after operation.

## Data Availability

The datasets used and analysed during the current study are available from the corresponding author on reasonable request.
